# Proposed Framework for Making Focused Antenatal Care Services Accessible: A Review of the Nigerian Setting

**DOI:** 10.5402/2011/253964

**Published:** 2011-12-29

**Authors:** John Ekabua, Kufre Ekabua, Charles Njoku

**Affiliations:** ^1^Department of Obstetrics and Gynaecology, University of Calabar Teaching Hospital, Calabar Cross River State, P.O. Box 2522 Calabar, Nigeria; ^2^Department of Community Medicine, University of Calabar Teaching Hospital, Calabar Cross River State, P.M.B. 1278 Calabar, Nigeria

## Abstract

The aim of this paper is to propose a framework for making antenatal care an effective strategy in reducing the high maternal mortality ratio in Nigeria. On-site visits to five teaching hospitals were carried out between 2006 and 2008 to assess the practice of antenatal care. Group discussions with 200 parturients on their awareness of birth preparedness/complication readiness were conducted, in October, 2008. The findings of this study are discussed in line with the proposed practice of focused antenatal care. The practice of antenatal care in all the hospitals visited was the traditional approach based on earlier European models. Awareness of birth preparedness/complication readiness, by parturients, as a goal-directed intervention during antenatal care was low (21.5%). To reduce maternal deaths through antenatal care, it is critical to link care with detecting and treating causes of maternal mortality by a skilled attendant.

## 1. Introduction

Antenatal care (ANC) is an umbrella term used to describe the medical procedures and care that are carried out during pregnancy [1]. It is the care a woman receives throughout her pregnancy and is important in helping to ensure a healthy pregnancy state and safe childbirth. The objective, therefore, of antenatal care is to assure that every wanted pregnancy results in the delivery of a healthy baby without impairing the mother's health [2]. Major goals of ANC are to

- promote and maintain the physical, mental, and social health of mother and baby by providing education on nutrition, personal hygiene, and birthing process;
- detect and manage complications during pregnancy, whether medical, surgical, or obstetrical;
- develop birth preparedness and complication readiness plan;
- help prepare mother to breastfeed successfully, experience normal puerperium, and take good care of the child physically, psychologically, and socially.

Organized antenatal care was introduced in the United States by social reformers and nurses in 1900 [3]. In a 1914 study by Williams, antenatal care reduced fetal mortality by 40% [4]. In the 1950s, Merkatz et al. observed that the most important factor responsible for improved maternal health was antenatal care [3]. By the 1980s, 75% of American women began antenatal care during the first trimester [5]. The United States Public Health Service (1992) goal for the year 2000 is for at least 90% of American women to begin antenatal care in the first trimester [6]. In 1986, the Department of Health and Human Services convened an expert panel to review the content of antenatal care [7]. Because health care during pregnancy depends on health before pregnancy, the panel recommended that preconception care should be an integral part of antenatal care [8, 9]. While antenatal care was developing in the United States, a similar movement also began in England in 1900 by the efforts of James Ballantyne [10, 11]. In Nigeria, the pioneering work of Lawson and Stewart in setting up organized maternity services in the 1950s and 60 s is commendable [12]. However, a dangerous trend developed in the 1990s with loss of faith in the system and increasing patronage of spiritual churches and unorthodox health facilities [13–15]. The aim of this study is to review the current practice of antenatal care and propose a framework for making antenatal care an effective strategy in reducing the high maternal mortality ratio in Nigeria. 

## 2. Method

On-site visits to five teaching hospitals and interaction with the resident doctors were carried out over a three-year period (2006–2008), to assess the practice of antenatal care. The teaching hospitals are University College Hospital Ibadan; Lagos University Teaching Hospital, Lagos; University of Nigeria Teaching Hospital, Enugu; Ahmadu Bello University Teaching Hospital, Zaria; University of Calabar Teaching Hospital, Calabar. Small group discussions with 20 obstetric registrars were held and four antenatal clinic sessions observed, in each hospital. A survey of 200 parturients in Calabar Municipality of Cross River State on their awareness of birth preparedness/complication readiness was conducted, from August to October, 2008. Review of relevant literature was done to see the place of antenatal care in reducing maternal mortality in a third world setting.

## 3. Results

In all the five teaching hospitals, antenatal care practice was based on the traditional European models developed in the early 1900s. Small group discussions revealed high level of awareness (80%) of tenets of focused antenatal care, among resident doctors. Birth preparedness/complication readiness as a goal-directed intervention was usually not discussed in up to 92% of antenatal clinic sessions observed, especially if the pregnant woman was considered healthy or “low risk.” Awareness of birth preparedness/complication readiness, as a goal-directed intervention during antenatal care was low (21.5%) among parturients surveyed in Cross River State, Nigeria.

## 4. Discussion

Nigeria is the most populous black nation on earth: out of every four Negroid one is a Nigerian, [16, 17]. Also, Nigeria makes up about 1% of world population but contributes 10% of global maternal mortality [18]. This demographic profile makes the issue of maternal mortality reduction in Nigeria a global concern [19]. Despite her endowment with great human and natural resources, Nigeria has continued to experience poor reproductive health indices with high maternal mortality ratios. ANC from a trained provider is important in monitoring pregnancy and helping to reduce the risks for the mother and child during this period. The present practice of antenatal care, as seen in the five tertiary health institutions, is based on European models of the 1900s, which emphasizes individualized care and risk assessment. The effect of this type of care is deterioration in utilization of maternity services and increasing maternal morbidity and mortality, in Nigeria [13–15]. The 2003 Nigeria Demographic Health Survey (NDHS) shows that six in ten mothers received antenatal care at least once from a trained medical professional [16, 17]. The most common antenatal care providers are nurses or midwives (37 percent). More than one-third of mothers (37 percent) did not receive any antenatal care. Among women who receive antenatal care, 17 percent make their first ANC visit during the first three months of pregnancy. According to the 2003 NDHS, only 58% of pregnant women received iron supplementation, 39% had drugs for malaria prevention, and 40% received two or more doses of tetanus toxoid. However, the data from the 2003 NDHS are limited to those areas surveyed and not representative of the true picture in many remote communities. Many women who are remote from survey sites experience several barriers to accessing health care. The scenario is not much different in other tropical countries [20, 21]. Reports from other centers have shown that, even when women attend ANC, they do not receive the full care as prescribed in national reproductive and child health programme (RCH/MCH) guidelines: 37% never had their blood pressure checked, 41% never had their blood tested, 45% never had their urine tested, 25% never had their abdomen examined, and 63% were never informed of any danger signs [22].

### 4.1. Problems Affecting Operation of Antenatal Care Services in Nigeria

There are many factors that act as barriers to effective antenatal care:

1. poor access—due to inadequate or nonexisting communication facilities [13];
2. poverty—services not affordable because of commercialization of health services and high cost of living [23];
3. prevailing cultural norm—where women need their spouses' consent before receiving care and the examination of a woman by a male obstetrician is not acceptable;
4. patient's perception of the quality of antenatal care services which is negatively affected by
  - prolonged outpatient waiting time,
  - increasing interventions, for example, induction of labour, caesarean section, and blood transfusions,
  - unprofessional conduct of service providers,
  - superstition with the trend of ascribing complications during pregnancy and labour to spiritual attack [13];
5. personnel cost in terms of poor remunerations and long hours of work;
6. paralysis of emergency obstetric care due to inadequate facilities on ground;
7. perennial power outage.

## 5. Proposed Organization of Antenatal Care Service

Women and newborns need timely access to skilled care during pregnancy, childbirth, and the postpartum/newborn period. Too often, however, their access to care is impeded by delays [24]. These delays have many causes, including logistical and financial concerns, unsupportive policies, and gaps in services, as well as inadequate community and family awareness and knowledge about maternal and newborn health issues [25].

 The setup of antenatal care in a tropical setting, like Nigeria, must be structured to meet the intended goal of reduction in maternal and child morbidity and mortality. For this to happen, antenatal care must be acceptable, accessible, and affordable to the community. At the primary health care level, the antenatal care facility should be

1. within a 15-minute walking distance from the furthest pregnant woman [14, 15];
2. manned by trained personnel who live within the community and are familiar with the norms of the area;
3. made up of well-defined but interrelated sections which include
  - the reception/waiting room,
  - the nurses station,
  - the examination/weighing room,
  - the records department/store,
  - a dispensary/side laboratory,
  - a consulting room for a visiting doctor from a supervising secondary health institution;
4. affiliation to a secondary health care facility which exercises oversight over groups of primary health facilities with antenatal clinics in the area;
5. equipped with portable water and sustainable power supply.

Minimum staff requirement for this level of antenatal care should include a midwife, receptionist/records clerk, orderlies and sanitation workers, and a laboratory assistant. A community health extension worker where available is an asset, in carrying out-home visits and assessing the environmental health needs of patient's residence. Also the introduction of home-based record system will facilitate the process of clinic visit and reduce waiting time.

 Within a county or local government area, secondary health facilities providing antenatal care should be sited within 20 kilometers of each other. Where population density is high, the distance between such facilities may be shorter to improve coverage. Antenatal clinics at the secondary level should provide service for all pregnant women in their area of coverage and for pregnant women with complications referred from the primary health care level. Each province, state, or geopolitical area should have a tertiary health facility providing antenatal care service. Care, preferably, should be limited to all pregnant women with medical and obstetric complications who register directly for ANC or are referred. This will justify the huge resources and personnel invested in these centers of excellence.

## 6. Practice of Antenatal Care

The traditional approach to antenatal care, which is based on European models, assumes that better care is achieved by frequent routine visits. Evidence-based research has found the practice of the traditional approach to ANC based on the European models to be wasteful and misleading [1]. Women are classified by risk status to determine their chances of complications and the levels of care needed. Many developing countries, like Nigeria, have adopted this approach without taking into account available resources and peculiar needs of their population. Risk approach is not an efficient or effective strategy for reduction of maternal mortality because risk factors cannot predict occurrence of complications. Also, majority of women considered low risk experienced complications, while most women considered high risk delivered without experiencing a complication [26, 27]. The Maternal and Neonatal Health (MNH) programme promotes the concept of focused antenatal care which emphasizes quality over quantity of visits [26]. This approach recognizes two keys facts.

1. Frequent visits do not necessarily improve pregnancy outcome and, in developing countries, are often logistically and financially impossible for most women.
2. Many women who have risk factors never develop complications, while women without risk factors often do. The implication is that scare health care resources may be devoted to unnecessary care for “high-risk” women who never develop complications, and “low-risk” women may be unprepared to recognize or respond to signs of complications.

Focused antenatal care recognizes that every pregnant woman is at risk for complications and should receive the same basic care and monitoring for complications [27]. Focused antenatal care approach is based on evidence-based interventions that address the most prevalent health issues that affect mothers and newborns [26]. The major goal of focused antenatal care is to help women maintain normal pregnancies through targeted assessment and individualized care [28]. The focus is on individualized care and not number of routine visits.

## 7. Goal-Oriented Interventions in Focused Antenatal Care

Goal-directed interventions give a framework for effective antenatal care. These include the following components.

1. Care from a skilled birth attendant and continuity of care.
2. Detection and early treatment of conditions that could severely affect maternal and fetal well-being such as HIV, syphilis and other sexual transmitted infections, malaria, tuberculosis, malnutrition and severe anemia, vaginal bleeding, hypertensive disorders, malpositions after 36 weeks, and fetal distress.
3. Preventive interventions which include
  - tetanus toxoid prophylaxis,
  - iron and folate supplementation,
  - intermittent preventive treatment for malaria,
  - presumptive treatment for hookworm,
  - vitamin A supplementation,
  - iodine supplementation,
4. Counseling and health promotion on
  - recognition of danger signs during pregnancy and labour and appropriate action to be taken,
  - importance of good nutrition,
  - risk of alcoholism and substance abuse,
  - adequate rest during pregnancy,
  - family planning,
  - breast-feeding,
  - HIV prevention.
5. Preparation for childbirth and complication readiness: the skilled provider and the woman should plan for the following:
  - a skill provider to be present at the birth,
  - the place of delivery and how to get there,
  - items needed for delivery,
  - need to save money in order to meet financial commitments/bills during childbirth,
  - support during and after childbirth,
  - appropriate response in case of life-threatening complications: this will include
    - a person designated to make decision on her behalf, in case she is indisposed,
    - a way to communicate with a source of help,
    - a source of emergency funds,
    - emergency transportation,
    - blood donors.

The implementation of focused antenatal care as elucidated above in this paper is far from being feasible in developing countries, like Nigeria, for the following reasons.

1. Lack of political will on policy change and payment of lip service by the powers that are on the issue of maternal and neonatal morbidity/mortality.
2. Diversion of state funds by politicians needed for infrastructural development to personal use/corruption.
3. Difficulty in changing the status quo in current medical practice (old habits die hard); from traditional practice of routine ANC to the practice of focused ANC.
4. Unwillingness of available trained health personnel to live in rural areas because of poor infrastructure/social amenities; flight of health personnel to greener pastures in industrialized nations.
5. Dearth of infrastructure/personnel for implementation of focused antenatal care.
6. Adverse economic climate/commercialization of health services.
7. Prevailing cultural norms, illiteracy and superstition that promote patronage of unskilled birth attendant/traditional healers.
8. Loss of faith in orthodox health care because of spiraling maternal and neonatal morbidity/mortality.

## 8. The Way Forward

There is need for a national rethink/rebirth. This can begin with the convening of a national conference on maternal and neonatal health. This conference should be sovereign and the decisions binding on all. It should also be all encompassing with intergovernmental/intersectorial participation. All stakeholders should be well represented from the politician to the peasant woman who may be a hewer of wood and a drawer of water, in the rural/remote areas. All shades of opinion must be considered and universal participation by all stakeholders in drawing up an acceptable and enduring health care reform programme encouraged, with particular emphasis on the tenets of focused antenatal care. Ownership of the programme by all stakeholders is necessary for successful implementation and continuity of focused antenatal care. Improvement in social condition can be achieved through systematic eradication of illiteracy and equitable distribution of national wealth. Training/retraining of health personnel needed for implementation of focused antenatal care should be embarked upon.

## 9. Conclusion

The signing of the millennium development goals in 2000 by heads of government has not resulted in the expected improvement in reproductive health indices in the tropical countries, like Nigeria [29]. Nigeria, the most populous black nation, makes up 1.7% of total world population yet contributes 10% of global maternal mortality [19]. The organization of antenatal care services in developing countries, like Nigeria, is bedeviled by operational and logistical problems, resulting in decreased consumer satisfaction, increasing patronage of unorthodox health facilities, spiraling maternal and fetal demise. There is need for political will to change the status quo. Apart from putting in place the required facilities and personnel for antenatal care, the practice of focused antenatal care may be one way of restoring faith in the reproductive health delivery system.
